# Atypical Hailey-Hailey disease: Report of a unique case and review of the literature

**DOI:** 10.1016/j.jdcr.2026.04.035

**Published:** 2026-04-24

**Authors:** Jessica Houpe, Anna Wanzenberg, Alison Grise, Rebecca Gibons, Andras Schaffer, Stephen K. Richardson

**Affiliations:** a Division of Dermatology, University of Central Florida College of Medicine/HCA Florida Healthcare GME Consortium, Tallahassee, Florida; b University of Central Florida College of Medicine, Orlando, Florida; c Florida State University College of Medicine, Tallahassee, Florida

**Keywords:** atypical, benign familial pemphigus, dermatology, Hailey-Hailey disease, segmental mosaicism, topical corticosteroid

## Introduction

Hailey-Hailey disease (HHD), or benign familial pemphigus, is an autosomal dominant acantholytic disorder caused by a mutation in the ATP2C1 gene that encodes a calcium-dependent ATPase (hSPCA1). This mutation disrupts the calcium gradient across keratinocytes resulting in desmosome dysfunction and acantholysis of the suprabasilar epidermis.^1^^,^^2^ Patients typically present with cutaneous lesions during the first 4 decades of life with no predilection for sex or race. Classically, lesions appear as crusted erosions and fissured plaques in areas prone to friction and sweating.^2^ The disease course is relapsing and remitting in nature. Additionally, it may be complicated by infection and, rarely, the development of squamous cell carcinoma. We describe a patient who presented with isolated lesions on her back as the sole manifestation of Hailey-Hailey disease, thus adding to the sparse but growing literature on atypical variants of this uncommon condition.

## Case report

A 61-year-old female with no significant medical history presented with multiple annular erythematous crusted plaques on her back that developed over a several week period. She reported identical self-limited eruptions in the same distribution almost a decade prior that resolved with the application of high-potency topical steroids. The lesions began as discrete papules that coalesced into annular plaques with expanding borders.

**Fig 1:**
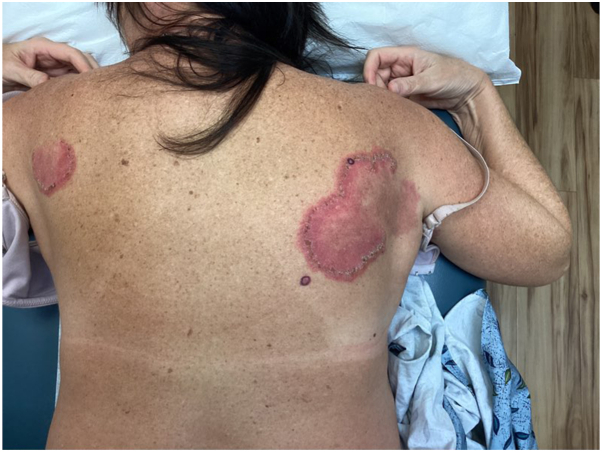
Expanding annular violaceous plaques on the back with eroded borders.

**Fig 2:**
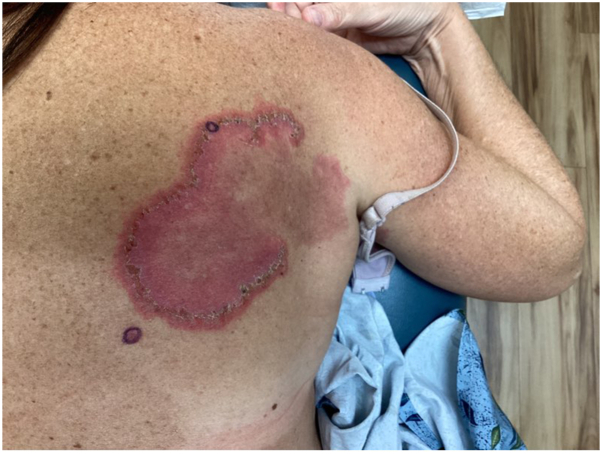
Representative violaceous plaque with central clearing and trailing border with serous crust.

She denied any history of intertriginous involvement or associated symptoms. Her presentation was notable for 2 discrete 8-12 cm annular violaceous-red plaques with adherent scale and a trailing erosive border with associated serous crust (Figs 1 and 2). Clinically, the differential diagnosis included blistering dermatoses (eg, pemphigus foliaceous [erythema annulare-type], pemphigus vulgaris); acantholytic diseases (e.g., HHD, Darier’s disease); figurate erythemas (eg, erythema annular centrifugum, subacute cutaneous lupus erythematosus); and cutaneous T cell lymphoma. Her son experienced similar lesions as a young adult that were also episodic in nature. She denied affected parents and had no recollection of other affected family members.

**Fig 3:**
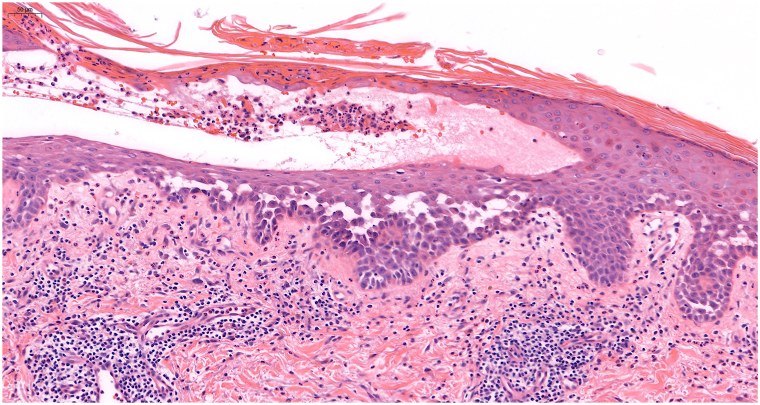
Intraepidermal vesicle with associated acantholysis.

**Fig 4:**
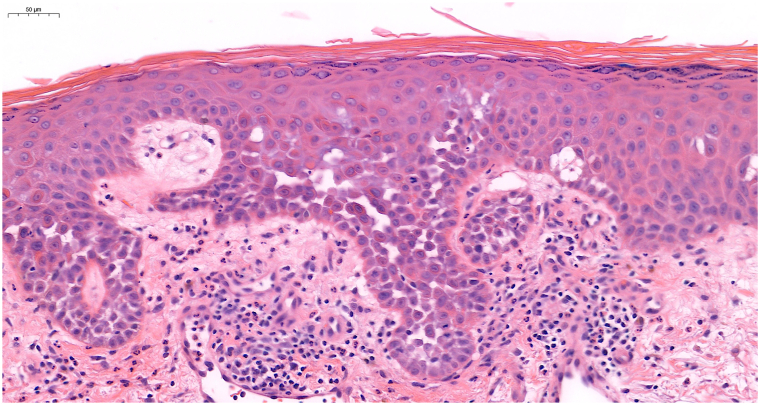
Keratinocyte acantholysis with “dilapidated brick wall” appearance.

A skin biopsy from the large plaque on her right upper back showed incomplete suprabasilar acantholysis mimicking a “dilapidated brick wall,” focal areas with intraepidermal vesicles, “tomb-stoning” of the basilar cells with adnexal involvement, and an absence of corps ronds and grains (Figs 3 and 4). Histologically, the differential diagnosis included acantholytic diseases such as HHD, Darier’s disease, and Grover’s disease; as well as blistering disorders affecting the suprabasilar layer (eg, pemphigus vulgaris). Direct immunofluorescence was negative and autoimmune bullous disease serologies were unremarkable, favoring a diagnosis of HHD.

The patient was treated with clobetasol 0.05% ointment twice daily for 2 weeks resulting in rapid clearing of her skin lesions.

## Discussion

1. We describe an atypical presentation of HHD localized to the back of an adult female without concurrent or prior intertriginous involvement supported by clinical findings, histology, immunofluorescence studies, and autoimmune bullous disease serologies. Classic histology of HHD shows scale crust, minimal dyskeratosis, suprabasilar acantholysis, and a “dilapidated brick wall” appearance; our case exhibited adnexal acantholysis, an atypical feature not commonly associated with HHD.^3^
2. The clinical differential diagnosis included pemphigus vulgaris which was ruled out by negative immunofluorescence studies and the absence of localized subrabasilar acantholysis.^4^
3. Atypical variants of HHD may present with segmental mosaicism, which are characterized by lesions distributed along lines of Blaschko.
  1. Type 1 mosaicism arises from an early post-zygotic somatic mutation that clinically manifests as unilateral segmental lesions.
  2. Type 2 mosaicism results from a new post-zygotic mutation that arises in the setting of a preexisting germ line mutation; these patients present with severe atypical forms of HHD segmentally superimposed on classic HHD sites, often with involvement of deep adnexal structures.^4^^,^^5^
4. In our literature review, we identified 29 cases of atypical HHD from 17 published studies (Supplementary Table I, available via Mendeley at https://data.mendeley.com/datasets/cxygbgcnhj/1).
  1. In atypical cases, the majority affected men (62% of cases) and the median age at diagnosis was 48 y versus classic HHD which equally affects men and women in their second to third decades of life.
  2. Family history was noted in 76% of cases with most patients experiencing lesions months to decades prior to their clinic presentation.^6^^,^^7^
  3. Only 24.1% of cases (*n*= 7) reported no history of intertriginous involvement.
5. Treatment is directed toward symptomatic relief, minimizing disease burden and reducing the duration of outbreaks.
  1. Our patient improved with the application of a high-potency topical steroid (clobetasol 0.05% ointment) for 2 weeks.
  2. Published reports support this approach in addition to intralesional/systemic steroids, antibiotics, intradermal botulinum toxin type A, dapsone, isotretinoin, and topical immunomodulators.^1^
  3. More recently, promising results have been achieved with dupilumab and topical ruxolitinib 1.5% cream twice daily.^8^
6. In conclusion, we describe an atypical case of HHD isolated to the back, with prominent adnexal involvement, and absence of preceding family history.
  1. Her history of an affected son and the absence of disease manifestations in her parents suggests a de novo germline mutation.
  2. Whether this constellation of findings represents a unique subtype of atypical HHD remains to be determined as more cases are identified and reported.
7. Dermatologists should be cognizant of uncommon presentations of HHD in clinical practice.

## Conflicts of interest

None disclosed.
